# Multiplex Assay to Determine Acute Phase Proteins in Modified Live PRRSV Vaccinated Pigs

**DOI:** 10.1021/acs.jproteome.4c00154

**Published:** 2024-07-15

**Authors:** Marc Tor, Lorenzo Fraile, Francisca Vilaró, Ramona N. Pena

**Affiliations:** † Animal Science Department, University of Lleida − Agrotecnio-CERCA Center, Lleida 25198, Spain; ‡ Scientific-Technical Services TCEM, Universitat de Lleida, Lleida 25198, Spain

**Keywords:** acute phase protein, selected reaction monitoring, porcine reproductive and respiratory syndrome

## Abstract

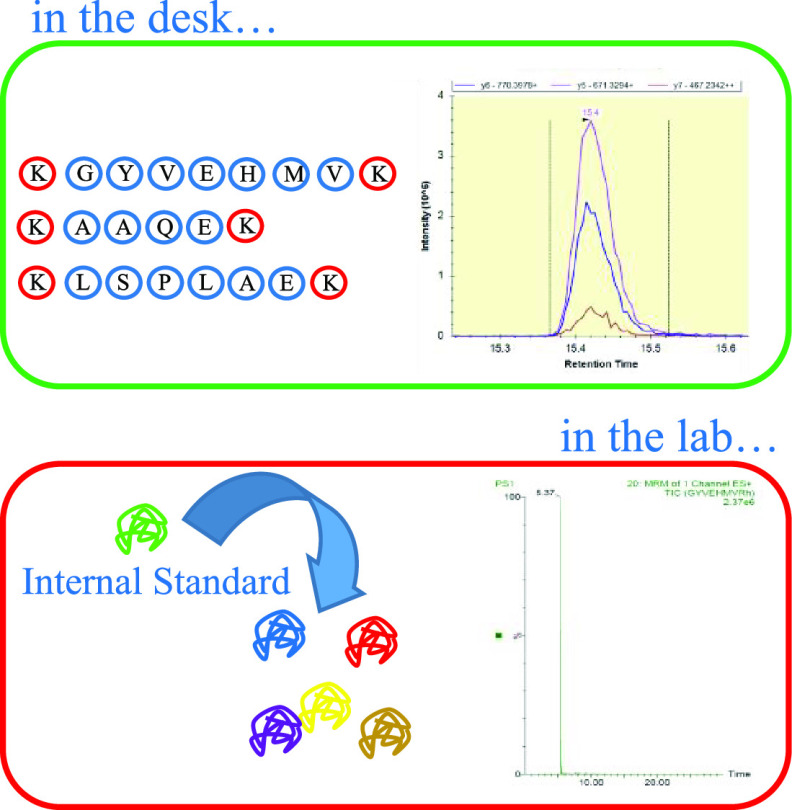

Acute phase protein (APP) response to vaccine challenges is an attractive alternative to natural infection for identifying pigs with increased disease resilience and monitoring the productive performance. Currently, the methods used for APP quantification are diverse and often based on techniques that use antibodies that are not necessarily pig specific. The objective of this work is the development of a method based on a UPLC-SRM/MS system for simultaneous determination of haptoglobin, apolipoprotein A1, C-reactive protein, pig-major acute protein, and serum amyloid A and its application in pigs to monitor the effect of a vaccine administered against porcine reproductive and respiratory syndrome virus (PRRSV). With the aim of tracing the complete analytical process for each proteotypic peptide, a synthetic QconCat polypeptide construct was designed. It was possible to develop an SRM method including haptoglobin, apolipoprotein A1, pig-MAP, and serum amyloid A1. The PRRSV vaccine only affected haptoglobin. The pigs with positive viremia tended to show higher values than negative pigs, reaching significant differences in the three haptoglobin SRM-detected peptides but not with the data acquired by immunoenzymatic and spectrophotometric assays. These results open the door to the use of SRM to accurately monitor APP changes in experimental pigs.

## 1 Introduction

Acute phase proteins (APPs) are plasma proteins whose concentration is related to inflammation and changes after trauma or infection. According to their response after a challenge, APP can be classified as positive APP when plasma concentration increases after an aggression, like haptoglobin (HP), pig-MAP, serum amyloid A (SAA), C-reactive protein (CRP), and alpha-2-HS-glycoprotein, or negative APP if their concentration drops, like apolipoprotein A1 (APOA1), serum albumin, or transthyretin. The magnitude of response of each APP differs according to the cause of reaction; e.g., different pathogens result in different APP responses.

APP analysis is useful both in veterinary diagnosis and in the monitoring of treatments and the evolution of diseases.^1^ It can also be applied in the monitoring of animal welfare as well as in the evaluation of the growth potential of production animals^2^ and even in the field of food safety.^3^ In pig breeding, one critical point to incorporate disease resilience into breeding programs is the ability to detect phenotypes with precision in the population. The APP response to vaccine challenges is an attractive alternative to natural infection for identifying pigs with increased disease resilience.^4^ The use of vaccines allows all animals to be vaccinated at the same dose/age/time, and thus, response phenotypes can be collected with higher consistency, and it may be used as an index for monitoring productive performance. Moreover, low serum APP after a challenge has been correlated with better production parameters in pigs,^5−7^ which makes them good candidate markers for immune phenotypes of resilience to infections.

Currently, the methods used for APP quantification are diverse and often based on techniques that use antibodies that are not necessarily pig specific. In addition, current protocols do not usually support the distinction between isoforms or the evaluation of post-translational modifications.^8^ Immunoenzymatic analysis methods may have specificity problems and can hardly be multiplexed^9^ because the antibodies' cross-reactivity cannot always be overcome, limiting assay performances and resulting in inaccurate results.^10^ An alternative to immunoenzymatic and spectrophotometric assays is the development of targeted proteomics protocols based on UPLC-SRM/MS (ultraperformance liquid chromatography-selected reaction monitoring mass spectrometry) methods. Using the available information derived from APP gene and protein sequences,^11^ many drawbacks of the immunoenzymatic and spectrophotometric assays could be overcome including the distinction between isoforms and post-translational modifications. In addition, the use of synthetic polypeptides constructed from proteotypic peptides could allow precise and simultaneous absolute quantification of multiple proteins and even proteomes in different species and tissues.^12−14^

Thus, with the aim of facilitating the use of APP in pig breeding programs, the objective of this work is to advance the development of a method based on a UPLC-SRM/MS system for simultaneous determination of pig plasmatic APP. First, the quantification of the profile of five plasma APPs was set up, including Haptoglobin, apolipoprotein A1, C-reactive protein, pig-major acute protein, and serum amyloid A. Once optimized, the method was cross-validated with established immunoenzymatic and spectrophotometric methods and applied to monitor the effect of a vaccine administered against PRRSV (porcine reproductive and respiratory syndrome virus) in pigs.

## 2 Materials and Methods

### 2.1 Reagents, Solvents, and Standards

Solvents (LC–MS grade) were purchased from Fisher Scientific (Loughborough, United Kingdom). Ultrapure water was provided by a Milli-Q system (Millipore, Milford, MA, USA). Formic acid was from Merck (Darmstadt, Germany). The ProteinWorks eXpress Digest kit was from Waters (Milford, MA, United States). The QconCAT synthetic protein was synthesized and purified by PolyQuant GmbH (Bad Abbach, Germany).

### 2.2 Animals and Blood Samples

Fourteen commercial male Duroc pigs were randomly chosen at 6–7 weeks of age from health status farm located in the northern part of Spain. These pigs came from a PRRSV-negative farm, and they were porcine circovirus type 2-negative and clinically healthy at the beginning of the experiment. Pigs received nonmedicated commercial feed ad libitum and had free access to drinking water. Animals were housed in an experimental farm (CEP, Torrelameu, Lleida, Spain), identified, ear-tagged, randomly distributed into three pens, ensuring a stock density of 1 m^2^ by animal, and balanced by weight (range 10–20 kg). After a 7 day acclimation period (day 0), pigs were vaccinated intramuscularly with a commercial PRRSV modified live vaccine as recommended by the manufacturer (Porcilis PRRS, MSD Animal Health). Blood samples were collected at 0, 3, 7, 10, 14, 21, 28, 35, and 42 days postvaccination (DPV). Body weight was collected at 0 and 42 DPV. Pigs were euthanized with an intravenous overdose of sodium pentobarbital at 42 DPV. This experimental protocol was approved by the Ethical Committee of the University of Lleida, with reference number CEEA 01-05/13. For this research work, samples at 0, 3, 7, and 14 DPV were used.

### 2.3 PRRSV Viremia Determination

PRRSV viremia was measured using a semiquantitative TaqMan PCR assay for PRRSV RNA. The PCR was performed as a routine diagnostic test by personnel of the Group of Sanejament Porci (GSP, Lleida, Spain). Briefly, total RNA was isolated from serum using the LSI MagVet Universal Isolation Kit (Thermo Fisher Scientific Inc.) in accordance with the manufacturer’s instructions. An internal positive control, “IPC PRRS”, was included within each sample and extracted according to manufacturing instructions to validate RNA extraction step. Samples were analyzed with the LSI VetMAX PRRSV EU/NA Kit (Life Technologies, Thermo Fisher Scientific Inc.). Viral RNA was amplified as a one-step reverse transcriptase (RT)-PCR according to kit instructions. Each 25 μL reaction contained 7 μL of RNA and 18 μL of PRRS EU/NA Mix from the kit. The RT-PCRs were carried out on a QST 7500 Real-Time PCR System (Life Technologies, Thermo Fisher Scientific Inc.) in a 96-well format according to the manufacturer’s recommendations (10 min at 45 °C, 10 min at 95 °C followed by 40 cycles of 15 s denaturation at 95 °C and 70 s annealing at 60 °C). For the construction of a standard curve, ranging from 10 to 106 copies/mL, serial dilutions of a template RNA were prepared and assayed along with the samples (provided in the LSI VetMAX PRRSV EU/NA Kit). The assay results were reported as the log10 of PRRSV RNA copies/mL relative to the standard curve. Because of the sensitivity of PCR, less than 10 copies (before log-transformation) were assumed to have negligible amounts of virus in the serum relative to the standard and were given a value of 1 (corresponding to a log-transformed value of 0).

### 2.4 Trypsin Enzymatic Digestion

Digestion of blood serum was carried out by using 35 μL samples that were denatured, reduced, alkylated, and digested by ProteinWorks eXpress Digest according to the manufacturer’s protocol. Samples were centrifuged for 15 min at 3000*g* (23 °C), and tryptic peptides were obtained by SPE extraction of 50 μL of digested serum by using an Oasis MCX 96-well μElution Plate (Waters, Milford, MA, USA).

### 2.5 Liquid Chromatography and Mass Spectrometry

An ultra-high-performance liquid chromatography system coupled to a XevoTQS triple quadrupole mass spectrometer (Waters, Milford, MA, USA) was used for the analysis. The system was equipped with an electrospray ionization source and an ACQUITY UPLC column (CSH C18, 2.1 × 150 mm, 1.7 μm particle size). The injection volume was 5 μL. Equilibration was performed in 100% of eluent A (Milli-Q water containing 0.1% formic acid). After injection, eluent B (acetonitrile containing 0.1% formic acid) was linearly increased to 50% at 25 min and to 30% at 30 min. The flow rate was kept constant at 300 μL/min. The ESI source was operated under positive ion mode. The operating conditions were set as follows: capillary voltage 3.3 kV, source temperature 150 °C, desolvation temperature 400 °C, cone gas flow 150 L/h, desolvation gas flow 900 L/h, and collision gas flow 0.15 mL/min.

### 2.6 UPLC-SRM/MS APP Assay Design

#### 2.6.1 APP Selection and “In Silico” Digestion

Once the set of target APP was defined, known transcripts were determined by using the available annotated pig proteome obtained in bulk from the FTP Ensembl site (ftp.ensembl.org) (file: Sus_scrofa.Sscrofa11.1.pep.all.fa; accessed on 2021/03/09) (Table S1). By using the Skyline software package (Version 20.2; MacCoss Lab, Univ. of Washington),^15^ each transcript was in silico digested by trypsin allowing one missed cleavage, a peptide length between 5 and 25 amino acids and with a cysteine *S*-carbamidomethylation as a fixed modification. Only type y ions were taken into account, allowing 2 and 3 charges for precursors and 1 and 2 charges for ions. The recording range of the instrument was set between 50 and 1800 *m*/*z* units. The full list of known transcripts and the in silico digestion results are summarized in Table 1.

**Table 1:** In Silico Digestion of Five Acute Phase Proteins (APP), Namely, Haptoglobin (HP), Pig-Major Acute Phase Protein (Pig-MAP), Serum Amyloid A (SAA), C-Reactive (CRP) Protein, and Apolipoprotein A-I (APOA1)

**APP**	**transcript name**	**amino acids**	**tryptic peptides**^a^	**fully shared peptides**^b^	**unique peptides**^c^	**MRM plausible transitions**^d^
HP	HP-01	322	18	17	1	211
	HP-02	354	19		2	237
pig-MAP	ITIH4-201	651	28	27	0	588
	ITIH4-202	893	41		1	795
	ITIH4-203	925	43		0	831
	ITIH4-204	897	41		2	874
	ITIH4-205	930	41		0	906
	ITIH4-206	921	42		0	0
	ITIH4-207	924	43		1	929
	ITIH4-208	976	41		1	945
SAA	SAA1-01	138	8	0	8	1042
	SAA1-02	118	5		0	1109
	SAA1-03	122	5		0	0
CRP	CRP-201	222	6	1	0	1191
	CRP-202	200	4		0	0
	CRP-203	103	2		0	1223
	CRP-204	259	8		2	1251
	CRP-205	222	6		0	0
	CRP-206	221	5		4	1301
	CRP-207	266	8		1	1313
APOA1	APOA1-201	265	13	8	5	1473
	APOA1-202	279	15		7	1601

a Digestion settings: enzyme trypsin, min length 8 aa, max length 25 aa.

b Shared peptides by all transcripts of a protein.

c Unique peptides for a given transcript.

d Transition settings: y ion types; precursor charges up to 3; ion charges up to 2.

#### 2.6.2 Selection of Peptides and Transitions

**Figure 1:**
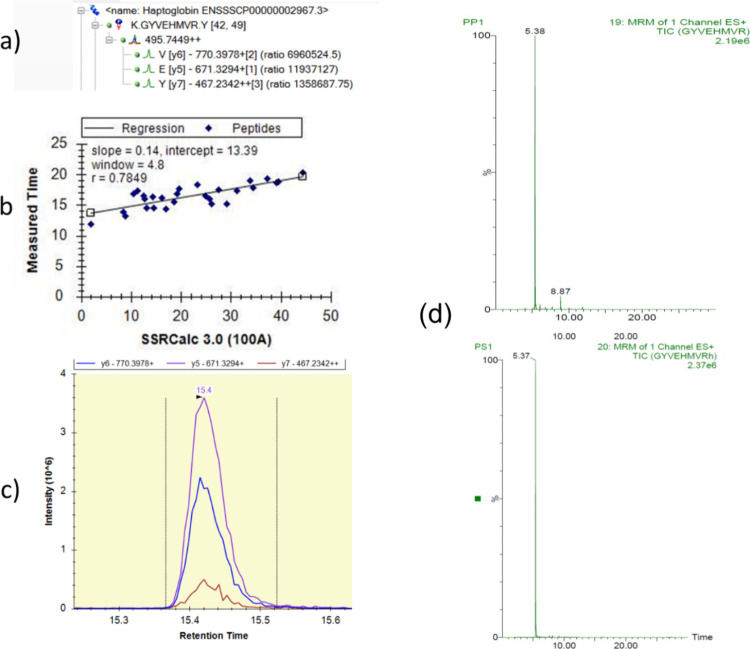
Selection and validation process of peptides and transitions for MRM method building. (a) Tryptic peptide plausible transitions generated by Skyline; (b) regression of real elution times and those estimated using the Sequence-Specific Retention Calculator 3.0 implemented on SkyLine; (c) coelution of three transitions of the GYVEHMVR peptide; and (d) signals of the natural transition (top) and the isotopically labeled (bottom) of the GYVEHMVR peptide. Matrix is blood plasma spiked with 0.376 pmol of QconCat.

The best peptides and transitions to be used for each transcript quantitation were experimentally selected by testing all 17,820 possible transitions (Table 1) in a pool of blood plasma from the same pigs under study. Taking into account that the transcripts of the same protein share many peptides, all possible transitions proposed in Table 1 could be placed in 50 SRM methods. Data refinement was performed in two stages: first through the Sequence-Specific Retention Calculator 3.0, implemented on SkyLine, establishing a threshold on the regression coefficient (*r*^2^ > 0.7) and eliminating the outliers, and second by visually confirming the coelution of at least three transitions. Figure 1a–c exemplifies the process with the peptide GYVEHMVR corresponding to the haptoglobin transcript ENSSSCT00000003046.4. Table 2 shows the peptides that passed these first two selection filters.

**Table 2:** Acute-Phase Protein (APP) Peptides Detected in a Pool of Samples of Pig Serum^a^

APP	name	fully shared peptides^b^	unique peptides^c^
HP	HP-01	K.GYVEHMVR.Y [42, 49]	0
	HP-02	R.YHCQTYYK.L [50, 57]	K.LPECEAVCGKPK.N [90, 101]
		R.IMGGSLDAK.G [78, 86]	
			
pig-MAP	ITIH4-201	R.FAHTVVTSR.V [48, 56]	0
	ITIH4-202	K.AAAQEQYSAAVAR.G [99, 111]	R.LTGPPGPLQPTR.F [637, 648]
	ITIH4-203	R.GESAGLVR.A [112, 119]	0
	ITIH4-204	K.VTFELVYEELLAR.H [140, 152]	R.VLPEVLSGATIPPPPAR.I [679, 695]
		R.HLGVYELLLK.I [153, 162]	
	ITIH4-205	R.FKPTLSQQQK.S [215, 224]	0
	ITIH4-206	R.DQFNLVSFSGEATQWK.K [308, 323]	0
	ITIH4-207	R.GQLHMENVTFVMESR.V [511, 525]	R.LTGSSVDPVFSHR.R [637, 649]
	ITIH4-208	R.VAEQEAEFLSPK.Y [526, 537] 27	0
			
SAA	SAA1-01		K.DSYSTLEAALR.G [85, 95]
			K.TQITSDLLACLR.N [115, 126]
	SAA1-02		0
	SAA1-03		0
			
CRP	CRP-201	K.ESENSYVSLTAR.L [33, 44]	0
	CRP-202		0
	CRP-203		0
	CRP-204		R.NDFQPFLK.R [31, 38]
	CRP-205		0
	CRP-206		K.GASVEAEASIILGQEQDTFAGEYEK.N [139, 163]
	CRP-207		0
			
APOA1	APOA1-201	K.VQPYLDDFQNK.W [120, 130] K.LSPLAEELR.D [164, 172]	K.EGGGSLAEYQAK.A [206, 217]
	APOA1-202		R.RPAPAHGRPLR.G [180, 190] R.VPGQGPGAAESAGR.E [202, 215] R.AGEPQGQHPGR.H [232, 242] R.RPPPALPSPSVR.L [256, 267]

a From the theoretical peptides proposed in Table 1, 28 plausible signals were detected in the pool serum sample. Within square brackets, the peptide position is indicated.

b Shared peptides by all transcripts of a protein.

c Unique peptides for a given transcript.

#### 2.6.3 Use of a Synthetic QconCAT Protein as a Reference for Chromatographic Retention Times and as an Internal Standard

With the aim of tracing the complete analytical process for each proteotypic peptide, a synthetic QconCat polypeptide was used as an internal standard. From the proteotypic peptides list, optimized for the UPLC/TQ equipment available in the SciTech Services of the University of Lleida (Table 2), a QconCat construct was designed, synthesized, and purified by PolyQuant GmbH (Table 3).

**Table 3:**
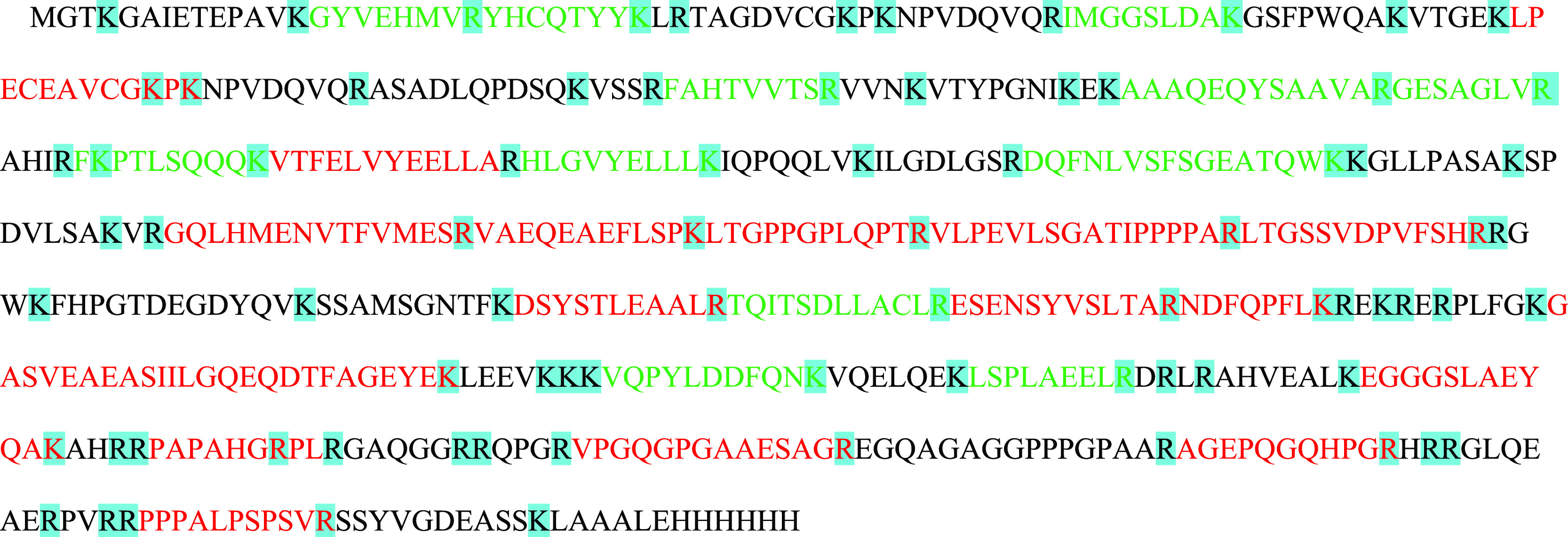
Synthetic QconCat Polypeptide Sequence^a^

a Green sequences are selected prototypic peptides whose retention time overlaps with that of the native peptide in blood plasma. Red sequences are selected proteotypic peptides whose retention time does not overlap with that of the native peptide in blood plasma. Black sequences are not proteotypic. Blue marks represent possible trypsin cleavage sites.

Beyond its use as an internal standard, the digestion and analysis of the QconCat construct also allow the comparison of the chromatograms of each proteotypic labeled peptide with its corresponding native peptide in blood plasma. This has allowed a third level of refinement in the validation of proteotypic peptides. If the retention time of the labeled peptide did not match the signal obtained in blood plasma from which that peptide was proposed, the peptide was not validated. Figure 1d) shows the case of the GYVEHMVR peptide as an example. It is clearly seen that the signal of the peptide in purity and the signal in blood plasma align perfectly, which corroborates, with a high level of certainty, that this peptide is being registered correctly. Table 3 indicates which peptides were validated and which were not by comparing their retention times.

A QconCat dilution series of 10 concentration levels across 3 orders of magnitude were constructed to determine the range of linearity and response for each studied peptide. In addition, each sample was spiked with 0.376 pmol of QconCat as an internal standard, allowing adjustment of the response curve sample by sample for each peptide. Results were processed using the QuanLynx software (MassLynx, Waters Corporation, Milford, MA, USA).

The intraday repeatability of the assay was monitored by analyzing in quintuplicate three blood samples from the same pig drawn on sampling days 0, 3, and 14. Thus, for each peptide, three different coefficients of variation were obtained, which represent a total of 36 coefficients of variation. These values are summarized in Figure S4.

### 2.7 Immunoassay and Spectrophotometric Analysis of APP

Serum haptoglobin was quantified by using a spectrophotometric method (hemoglobin binding assay) with the Tridelta PHASE Haptoglobin Assay (Tridelta Development Ltd., Ireland) performed on an automated analyzer (Olympus AU400, Hamburg, Germany). Intra-assay and interassay coefficients of variation for this technique have been reported previously.^3^ Serum pig-MAP was determined by a turbidimetric immunoassay with the TRUBOVET Pig-AMP kit (Acuvet Biotech, Zaragoza, Spain). Serum amyloid A1 was measured by a sandwich ELISA test with the Serum Amyloid A Enzyme Immunoassay (Tridelta Development Ltd., Ireland) using the EMS Reader MF V.2.9-0 (Labsystems, Helsinki, Finland).

### 2.8 Statistical Analysis

A linear regression model was used to cross-validate analytical methods. Because APP data present extreme values, correlation studies were performed using the nonparametric Spearman correlation coefficient. The APP and proteotypic peptides were analyzed using a mixed model. The model included the animal as a random effect, the day post vaccination (four levels: 0, 3, 7, and 14), the PCR diagnosis (two levels: positive and negative), and the interaction between day and PCR result as fixed effects. Fixed effects were tested using an *F* test. Multiple pairwise comparisons were tested using Student’s *t* test. All analyses were performed using the JMP 12.0.1 statistical software (SAS Institute Inc., Cary, NC, USA).

## 3 Results and Discussion

### 3.1 In Silico Digestion of the APP Set

Table 1 shows the known transcripts for the genes encoding the APP included in this study. Tryptic peptides were obtained by in silico digestion. Fully shared peptides are those present in all the transcripts of the same protein. Unique peptides are specific to a single transcript. The sum of the fully shared peptides and the specific peptides does not match with the total number of tryptic peptides because there are peptides that are not shared by all the transcripts, but neither are they specific because they are shared by more than one. Finally, the most probable transitions in the UPLC/TQ system to be used were obtained. As expected, the longer the protein amino acid chain is, the greater is the number of peptides, and therefore, the probability of finding transitions with a clear signal that can be quantified is also higher. Thus, from pig-MAP, the heaviest of the proteins studied, 27 peptides fully shared for all transcripts were obtained. Additionally, one transcript (ITIH4-204) had two unique peptides, three transcripts (ITIH4-202, ITIH4-207, ITIH4-208) had one unique peptide, and the rest of the transcripts did not have any unique peptides. In the case of haptoglobin, 17 fully shared peptides were found plus one unique peptide for the HP-01 transcript and two for HP-02. For apolipoprotein A1, eight fully shared and five and seven unique peptides were found for the APOA1-201 and APOA1-202 transcripts, respectively. In the case of the C-reactive protein, only one fully shared peptide and two, four, and one unique peptides were obtained for the CRP-204, CRP-206, and CRP-207 transcripts, respectively. In the case of the serum amyloid A1, the smallest protein studied, no fully shared peptides were found. Eight unique peptides were obtained for the SAA1-01 transcript, and five peptides were fully shared by the SAA1-02 and SAA1-03 transcripts.

With the aim of obtaining, on the one hand, peptides that could quantify all transcripts and, on the other hand, peptides that could distinguish each transcript specifically, the design focused on fully shared and unique peptides. This means that nonunique peptides that are only shared by some transcripts were not further considered. Because in the end many peptides could not be validated and the SRM method was not saturated, it might have been interesting to recover the partially shared peptides. This would be especially relevant for the serum amyloid A1, where fully shared peptides could not be validated (see below and Table 2). This is a clear work path for further development of the method.

### 3.2 Determination of Proteotypic Peptides

Once the theoretical transitions obtained were tested on a pool of blood plasma, Table 2 presents those peptides for which a plausible signal was obtained under our conditions. From the 88 theoretical peptides proposed, 28 plausible signals were obtained. Thus, nine, three, two, and one fully shared peptides were registered for pig-MAP, haptoglobin, apolipoprotein A1, and C-reactive protein, respectively. No fully shared peptides were registered for serum amyloid A1. In addition, signals were obtained for three unique peptides from pig-MAP, one from haptoglobin, five from apolipoprotein A1, and two from C-reactive protein and serum amyloid A1. This has been the basis for the design and assembling of the QconCAT synthetic protein.

### 3.3 Validation of Peptides by Using the QconCAT Synthetic Protein

Table 3 shows the amino acid sequence of the synthetic polypeptide QconCAT, which contains all the fully shared and unique peptides that were detected. In addition to using it as an internal standard to control the reproducibility of the enzymatic digestion, it can also be used as an external standard to locate the retention time of each peptide. This has been the third level of filtering to ensure that the signal registered in the sample really corresponded to the proposed peptide. Table 3 shows that 12 peptides (sequence in green) passed this last level of filtering, whereas 16 (sequence in red) did not. In summary, for haptoglobin and pig-MAP, only the complete validation of fully shared, but not unique, peptides has been possible. In the case of serum amyloid A1, only a unique peptide of the SAA1-01 transcript has been validated. In the case of the C-reactive protein, no peptides passed the last validation step. For apolipoprotein A1, it has been possible to validate two fully shared peptides.

Regarding intraday repeatability, Figure S4 summarizes the 36 variation coefficients obtained, 3 for each validated peptide. Most of them (∼70%) have values below 25%. However, two of them, both corresponding to sampling day 14, present exorbitant values. It is difficult to attribute this lack of repeatability to a specific step in the analytical process because the replicas include the enzymatic digestion process from the beginning. In view of the possible use of this method in the future by diagnostic laboratories, it would be advisable to also evaluate the interday repeatability through controls and make the samples in duplicate to detect possible mistakes.

**Figure 2:**
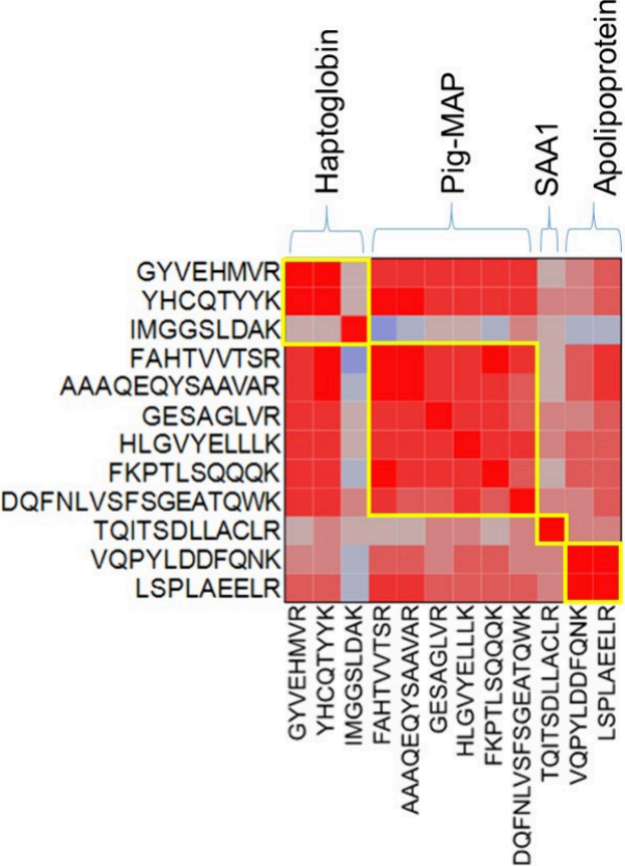
Color map of Spearman correlations for the 13 validated peptides. Yellow boxes group the proteotypic peptides from the same protein.

Figure 2 and Table S2 show the Spearman correlation coefficients for these 12 validated peptides. In the color map, in the first diagonal boxed in yellow, are the peptides from the same protein. Because they should present stoichiometric relationships, the correlation coefficients between them are very high, with values close to 0.9 for the fully shared peptides of each protein, except for the IMGGSLDAK peptide from haptoglobin, which, even though it is a fully shared peptide, correlates poorly with the other two validated fully shared peptides. In the case of apolipoprotein A1, the two fully shared peptides correlate well. In general, a high positive correlation is observed between the fully shared peptides of haptoglobin and pig-MAP, which agrees with the high correlation values (*r* = 0.79; *p* < 0.0001) between the two proteins when analyzed by classical immunoenzymatic and spectrophotometric techniques.

### 3.4 Cross-Validation with Immunoenzymatic and Spectrophotometric Methods

**Figure 3:**
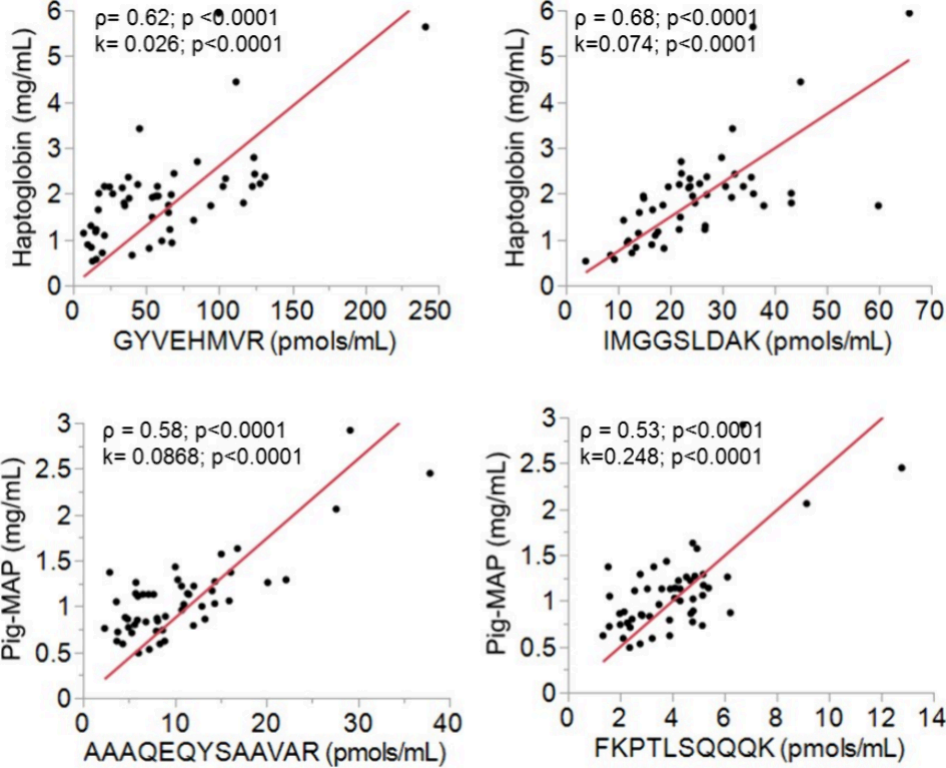
Comparison of haptoglobin and pig-MAP serum values determined by immunoenzymatic and spectrophotometric and MRM methods. ρ is the nonparametric Spearman correlation coefficient. *k* is the slope of the model that can be interpreted as the correction factor between both methods.

Table 4 shows the values of the APP determined immunoenzymatically or spectrophotometrically and the values of the corresponding proteotypic peptides. In the case of haptoglobin, pig-MAP, and serum amyloid A1, quantification was achieved both by enzymatic methods and by SRM. Therefore, it was possible to cross-validate both methods (Tables S3 and S4), although in the case of the SAA, the validation has not been possible because of a lack of significant correlation between data sets (Table S5). Haptoglobin and pig-MAP showed a significant positive relationship between the fully shared peptides and the immunoenzymatic and spectrophotometric values. Figure 3 presents the regression graphs and the Spearman correlation values. In the case of haptoglobin, it is striking that the values obtained by spectrophotometric assay present a good correlation with both the GYVEHMVR peptide and the IMGGSLDAK even though the correlation between the two peptides is poor (*r* = 0.13).

**Table 4:** Blood Concentration Values of Acute Phase Proteins Determined by Immunoenzymatic and Spectrophotometric Methods and Their Proteotypic Peptides Determined by UPLC-SRM/MS

	mean	standard error	CV
haptoglobin (mg/mL)	1.94	0.150	55.26
GYVEHMVR (pmol/mL)	55.56	5.065	75.71
YHCQTYYK (pmol/mL)	49.89	4.924	81.98
IMGGSLDAK (pmol/mL)	28.99	1.683	48.24
pig-MAP (mg/mL)	1.08	0.063	41.66
FAHTVVTSR (pmol/mL)	2.76	0.240	72.13
AAAQEQYSAAVAR (pmol/mL)	9.22	0.802	72.29
GESAGLVR (pmol/mL)	1.73	0.125	60.00
HLGVYELLLK (pmol/mL)	6.26	0.464	61.61
FKPTLSQQQK (pmol/mL)	3.69	0.228	51.23
DQFNLVSFSGEATQWK (pmol/mL)	7.66	0.792	85.83
SAA (μg/mL)	23.26	7.925	243.25
TQITSDLLACLR (pmol/mL)	5.05	0.199	32.74
CRP (mg/L)	22.62	2.267	72.95
VQPYLDDFQNK (pmol/mL)	50.86	1.629	26.61
LSPLAEELR (pmol/mL)	40.88	1.242	25.25

The initial units generated in the laboratory (mg/mL and pmol/mL for the immunoenzymatic or spectrophotometric and SRM methods, respectively) were maintained in the statistical analysis, as the proportion of each transcript is unknown, impairing the calculation of the molecular weight that should be used in the conversion of the units. In fact, if there were different proportions of transcripts of an APP in different animals, there could be a drop in the correlation coefficient between both methods. Regarding the order of magnitude of the values obtained by SRM, it is observed that the stoichiometric relationships are not always maintained.

Taking haptoglobin as an example, the mean values are 55.56 ± 5.06, 49.89 ± 4.92, and 28.99 ± 1.68 pmol/mL for the peptides GYVEHMVR, YHCQTYYK, and IMGGSLDAK, respectively (Table 4). The first two peptides show equivalent values and are very close to the spectrophotometric values of haptoglobin (calculated by taking the average value of the molecular weight of the two transcripts). In contrast, the IMGGSLDAK peptide behaves in a totally different way, presenting values close to 50%, although showing a good correlation with spectrophotometrically determined haptoglobin but not with the other two peptides.

The reason for this is not clear because all three are fully shared peptides. A plausible explanation would be the hypothesis that the haptoglobin transcription model is not yet complete and that there are other transcripts, yet unknown, that do not contain the IMGGSLDAK peptide. Because the SRM methods are based on information from the genome and proteome, as this information in pig is frequently updated, a refinement of the analytical methods might be necessary.

### 5.5 Effect of the PRRSV Vaccine on APP

We have previously shown that the response to a PRRSV modified live vaccine can be used to classify pigs according to their resilience capacity to this viral infection.^16^ Thus, pigs that do not raise a viremia (PCR-negative) after 21 DPV are classified as resilient, whereas those with PCR-positive results are considered susceptible to the infection. This classification has been shown useful, for instance, to predict the reproductive performance of sows undergoing PRRSV infection^17^ with positive economic effect when selected for.^18^ We hypothesized that resilient responses should be associated to low or moderate increases in positive APP such as haptoglobin, pig-MAP, C-reactive protein, and serum-amyloid A1 when compared to susceptible responses. The reverse would be expected for apolipoprotein A1, a negative APP.

**Figure 4:**
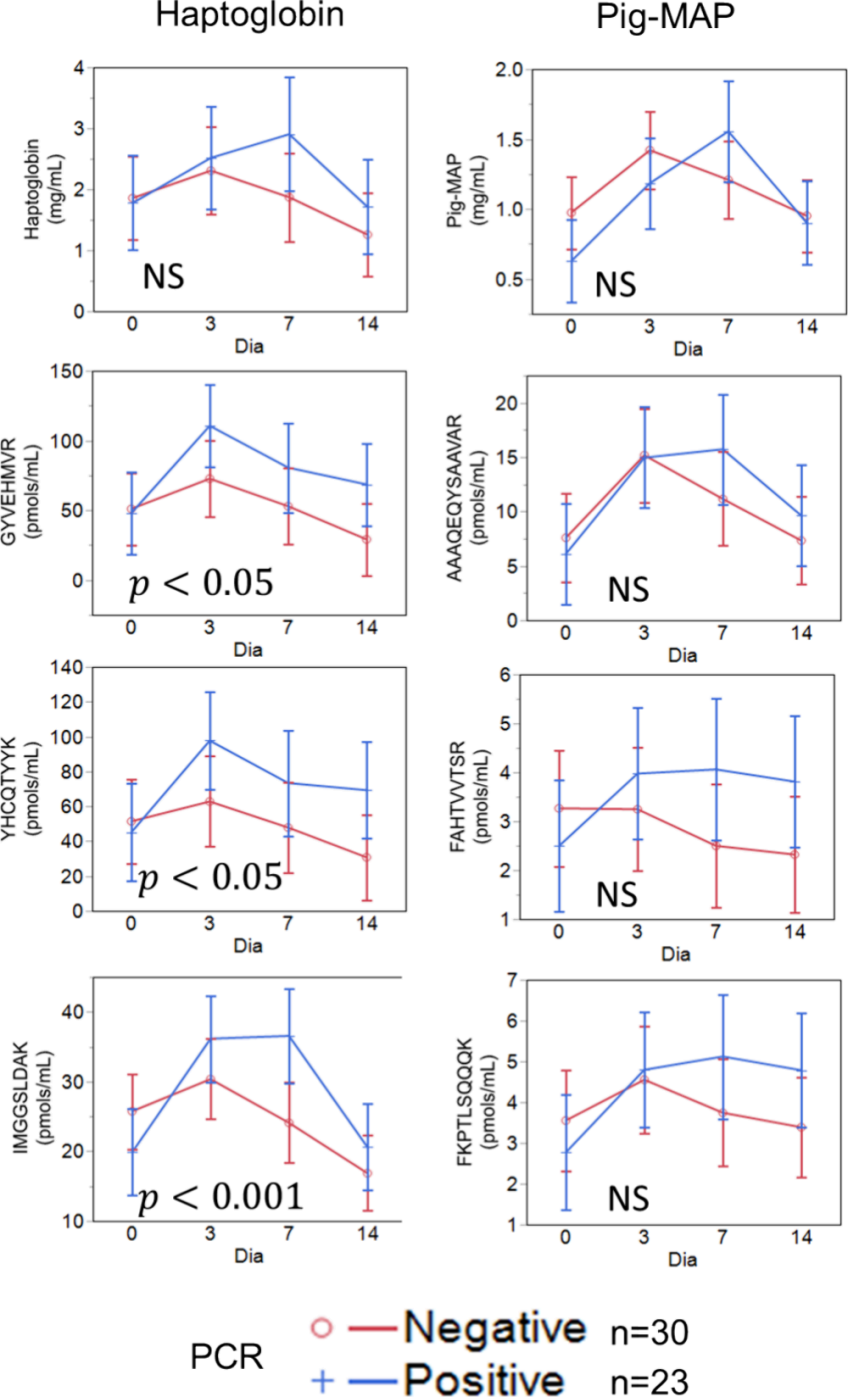
Haptoglobin and pig-MAP response to the PRRSV vaccine of pigs with negative and positive PCR results. Top panels, spectrophotometric and immunoenzymatic data; other panels, mass spectrometry data from validated peptides. Error bars represent standard error of the mean. *P* values correspond to differences between negative and positive groups.

Figure 4 shows the evolution of haptoglobin and pig-MAP up to 14 days after PRRSV vaccination in pigs with positive or negative PCR assays (positive or negative viremia). For both proteins, the first row corresponds to the APP values determined by immunoenzymatic and spectrophotometric assays and the following ones to the top three peptides determined by SRM. The trend of the haptoglobin evolution curve is similar whether it is determined by spectrophotometric assay or SRM, showing low values on the day of vaccination, a maximum on day 3 DPV, and then a gradual drop to return to the initial values by 14 DPV. The PCR-negative group tends to show higher APP values than the PCR-positive group, reaching significant differences in the three haptoglobin SRM-detected peptides but not with the data acquired by immunoenzymatic and spectrophotometric assays. Thus, the three haptoglobin peptides—GYVEHMVR, YHCQTYYK, and IMGGSLDAK—present significantly higher values in PCR-positive animals. The result was consistent for the three peptides analyzed. In the case of the pig-MAP, even though the shape of the curve resembles that of haptoglobin, there are no differences between PCR-positive and -negative animals either for the values determined by immunoenzymatic and spectrophotometric assays or for any of the peptides determined by SRM.

The C-reactive protein analyzed by means of antibodies shows a day effect with a maximum on day 3 DPV, but there were no differences between positive and negative animals (Figure S1). The SAA, measured both with antibodies and by its proteotypic peptide TQITSDLLACLR, did not present any significant effect with the studied model (Figure S2). No significant effect was obtained in the three proteotypic peptides of apolipoprotein, VQPYLDDFQNK, and LSPLAEELR (Figure S3). Taken together, these results indicate that the accurate measurement of distinct APP peptides can give additional information to the determination of APP, including data on the robustness of measurement by independent all transcript-shared peptides.

A previous research work has also confirmed that an acute phase response does exist in PRRSV-infected pigs,^19^ and other studies on natural PRRSV infection have shown similar APP dynamics compared with our results coming from vaccinated animals with a modified live vaccine. Thus, 5 week-old pigs infected with PRRSV displayed high values for haptoglobin and C-reactive protein, whereas pig-MAP did not differ statistically from noninfected controls.^20^ Also, in conventional herds with chronic PRRSV infection, pigs showed elevated haptoglobin and C-reactive protein serum concentrations.^21^ In summary, among the different acute phase proteins, haptoglobin was the most sensitive biomarker for PRRSV infection, C-reactive protein behaved in general as moderate (lower increases in serum concentration), and pig-MAP was the least responsive during the course of PRRSV experimental infection. Curiously, haptoglobin was also better than C-reactive protein or pig-MAP for Aujeszky disease.^20^ On the other hand, pig-MAP has been suggested as a better marker than haptoglobin for PCV2-SD,^22^ whereas haptoglobin and C-reactive protein showed a similar behavior for this viral infection. Finally, previously published research has shown an increase in acute phase proteins following the vaccination of lambs and pigs with different species-specific pathogens.^23,24^ These works report an increase in HP, CRP, and SAA in the period prior to 3 days post treatment, which was the initial point of study of the present work. This suggests the possibility of going deeper into the present work by determining the evolution of said APPs during the first 3 days after treatment.

## 4 Conclusions

In this work, an SRM method has been developed to quantify five APP in pig serum samples, including haptoglobin, apolipoprotein A1, pig-MAP, and serum amyloid A1. However, it has not been possible to find any proteotypic peptide for the C-reactive protein. Of the four proteins included, two have been validated with immunoenzymatic and spectrophotometric methods, obtaining moderate correlation coefficients, i.e., between 0.5 and 0.7 for haptoglobin and between 0.5 and 0.6 for pig-MAP. Even so, from this work, it cannot be concluded whether both methods are equivalent or if the estimation is more accurate in any of the methods. However, the SRM technologies allow the measurement of more than one peptide per protein, including peptides common to all the transcript variants, which can result in more robust repeatability data.

Because SRM design relies on annotated genomic and proteomic information, the methodology can be refined if new relevant annotation updates are provided. The set of peptides that are neither fully shared nor unique has not been explored in the present work, and their study could bring additional information to complement the data acquired by other individual peptides.

## Data Availability

The data set supporting the results of this article is available in Peptide Atlas: Identifier: PASS05868. Data set type: SRM. Data set tag: APPsDUROC. Data set title: Acute phase proteins from porcine plasma.

## Supporting Information Available

Protein accession numbers (Table S1); Spearman’s correlation coefficients (Tables S2 to S5); nonsignificant APP responses to PRRSV (Figures S1 to S3); and distribution of the coefficients of variation (Figure S4) (PDF)

## ABBREVIATIONS

APP: acute phase protein
PRRSV: porcine reproductive and respiratory syndrome virus
HP: haptoglibin
SAA: serum amyloid A
CRP: C-reactive protein
APOA1: apolipoprotein A1
DPV: days postvaccination
