# Supplementary material for: Multiplex Assay to Determine Acute Phase Proteins in Modified Live PRRSV Vaccinated Pigs
Source: J Proteome Res. 2024 Jul 15;23(8):3515–23. doi: 10.1021/acs.jproteome.4c00154 (PMC11301773; doi:10.1021/acs.jproteome.4c00154)

Multiplex assay to determine acute phase proteins in modified live PRRSV vaccinated pigs.

Marc Tor[1], Lorenzo Fraile[1], Francisca Vilaró[2] and Ramona N. Pena[1].*

[1] Animal Science Department, University of Lleida – Agrotecnio-CERCA Center, 25198 Lleida, Spain; [2] Scientific-Technical Services TCEM, Universitat de Lleida, 25198 Lleida, Spain.

[*] Email corresponding autor: marc.tor@udl.cat

Table of contents.

Tables

Table S1. Protein Accession number list from available annotated pig proteome.

Table S2. Full list of Spearman's correlation coefficients and significance probability between 12 validated proteotypic peptides.

Table S3. Full list of Spearman's correlation coefficients and significance between immunoenzimatic determination and proteotypic peptides of Haptoglobin.

Table S4. Full list of Spearman's correlation coefficients and significance between immunoenzimatic determination and proteotypic peptides of Pig-MAP.

Table S5. Full list of Spearman's correlation coefficients and significance between immunoenzimatic determination and proteotypic peptides of Serum Amyloid A.

Figures

Figure S1: C-reactive reponse to PRRSV vaccine of pigs with negative and positive PCR results.

Figure S2: Serum Amyloid A reponse to PRRSV vaccine of pigs with negative and positive PCR results.

Figure S3: Apolipoprotein reponse to PRRSV vaccine of pigs with negative and positive PCR results.

Figure S4: Distribution of the coefficients of variation of the 12 validated peptides.

Table S1. Protein Accession number list from available annotated pig proteome.

APP	Accesion number[1]	Transcript Name	Amino acids
Haptoglobin	ENSSSCT00000003046.4	HP-01	322
	ENSSSCT00000039122.2	HP-02	354
Pig-MAP	ENSSSCT00000088757.1	ITIH4-201	651
	ENSSSCT00000069224.1	ITIH4-202	893
	ENSSSCT00000068769.1	ITIH4-203	925
	ENSSSCT00000061383.2	ITIH4-204	897
	ENSSSCT00000077998.1	ITIH4-205	930
	ENSSSCT00000038206.2	ITIH4-206	921
	ENSSSCT00000012534.4	ITIH4-207	924
	ENSSSCT00000065989.2	ITIH4-208	976
Serum Amyloid A1	ENSSSCT00000069689.1	SAA1-01	138
	ENSSSCT00000014604.4	SAA1-02	118
	ENSSSCT00000057003.2	SAA1-03	122
C-Reactive protein	ENSSSCT00000007016.4	CRP-201	222
	ENSSSCT00000054270.2	CRP-202	200
	ENSSSCT00000067758.1	CRP-203	103
	ENSSSCT00000072472.1	CRP-204	259
	ENSSSCT00000076706.1	CRP-205	222
	ENSSSCT00000083957.1	CRP-206	221
	ENSSSCT00000085437.1	CRP-207	266
Apolipoprotein A1	ENSSSCT00000034599.3	APOA1-201	265
	ENSSSCT00000053994.2	APOA1-202	279

[1] Obtained in bulk from FTP Ensembl site (ftp.ensembl.org) (File: Sus_scrofa.Sscrofa11.1.pep.all.fa; accessed on 2021/03/09).

Table S2. Full list of Spearman's correlation coefficients and significance probability between 12 validated proteotypic peptides.

	YHCQTYYK	IMGGSLDAK	FAHTVVTSR	AAAQEQYSAAVAR	GESAGLVR	HLGVYELLLK	FKPTLSQQQK	DQFNLSGEATK	TQITSDLLACLR	VQPYLDDFQNK	LSPLAEELR
GYVEHMVR	0.980<br>p<.0001	0.130<br>p=0.287	0.845<br>p<.0001	0.827<br>p<.0001	0.674<br>p<.0001	0.791<br>p<.0001	0.739<br>p<.0001	0.647<br>p<.0001	0.172<br>p=0.157	0.232<br>p=0.054	0.331<br>p=0.005
YHCQTYYK		0.040<br>p=0.744	0.895<br>p<.0001	0.813<br>p<.0001	0.716<br>p<.0001	0.802<br>p<.0001	0.776<br>p<.0001	0.654<br>p<.0001	0.193<br>p=0.112	0.260<br>p=0.030	0.372<br>p=0.001
IMGGSLDAK			-0.113<br>p=0.354	0.113<br>p=0.352	0.182<br>p=0.133	0.227<br>p=0.059	0.117<br>p=0.336	0.345<br>p=0.003	0.021<br>p=0.858	-0.174<br>p=0.151	-0.123<br>p=0.312
FAHTVVTSR				0.798<br>p<.0001	0.797<br>p<.0001	0.778<br>p<.0001	0.850<br>p<.0001	0.647<br>p<.0001	0.188<br>p=0.120	0.312<br>p=0.008	0.386<br>p=0.001
AAAQEQYSAAVAR					0.694<br>p<.0001	0.874<br>p<.0001	0.832<br>p<.0001	0.625<br>p<.0001	0.101<br>p=0.407	0.342<br>p=0.004	0.397<br>p=0.0007
GESAGLVR						0.716<br>p<.0001	0.819<br>p<.0001	0.693<br>p<.0001	0.259<br>p=0.031	0.135<br>p=0.266	0.253<br>p=0.035
HLGVYELLLK							0.808<br>p<.0001	0.804<br>p<.0001	0.196<br>p=0.105	0.369<br>p=0.001	0.392<br>p=0.0009
FKPTLSQQQK								0.685<br>p<.0001	0.157<br>p=0.196	0.297<br>p=0.013	0.340<br>p=0.004
DQFNLSGEATK									0.270<br>p=0.024	0.190<br>p=0.116	0.202<br>p=0.095
TQITSDLLACLR										0.257<br>p=0.032	0.175<br>p=0.149
VQPYLDDFQNK											0.732<br>p<.0001
LSPLAEELR											

Table S3. Full list of Spearman's correlation coefficients and significance between immunoenzimatic determination and proteotypic peptides of Haptoglobin.

Haptoglobin<br>(mg/mL)	0.545<br>p<.0001	0.492<br>p=0.0002	0.671<br>p<.0001
	GYVEHMVR<br>(pmols/mL)	0.9805<br>p<.0001	0.13<br>p=0.287
		YHCQTYYK<br>(pmols/mL)	0.04<br>p=0.744
			IMGGSLDAK<br>(pmols/mL)

Table S4. Full list of Spearman's correlation coefficients and significance between immunoenzimatic determination and proteotypic peptides of Pig-MAP.

Pig-MAP<br>(mg/mL)	0.541<br>p<.0001	0.782<br>p<.0001	0.579<br>p<.0001	0.654<br>p<.0001	0.687<br>p<.0001	0.512<br>p<.0001
	FAHTVVTSR<br>(pmols/mL)	0.798<br>p<.0001	0.797<br>p<.0001	0.778<br>p<.0001	0.850<br>p<.0001	0.647<br>p<.0001
		AAAQEQYSAAVAR<br>(pmols/mL)	0.694<br>p<.0001	0.874<br>p<.0001	0.832<br>p<.0001	0.625<br>p<.0001
			GESAGLVR<br>(pmols/mL)	0.716<br>p<.0001	0.819<br>p<.0001	0.693<br>p<.0001
				HLGVYELLLK<br>(pmols/mL)	0.808<br>p<.0001	0.804<br>p<.0001
					FKPTLSQQQK<br>(pmols/mL)	0.685<br>p<.0001
						DQFNLVSFSGEATQWK<br>(pmols/mL)

Table S5. Full list of Spearman's correlation coefficients and significance between immunoenzimatic determination and proteotypic peptides of Serum Amyloid A.

SAA (ug/mL)	0.038 p=0.786
	TQITSDLLACLR (pmols/mL)

Figure S1: C-reactive reponse to PRRSV vaccine of pigs with negative and positive PCR results. Only available immunoenzymatic data; Error bars represent standard error of the mean.

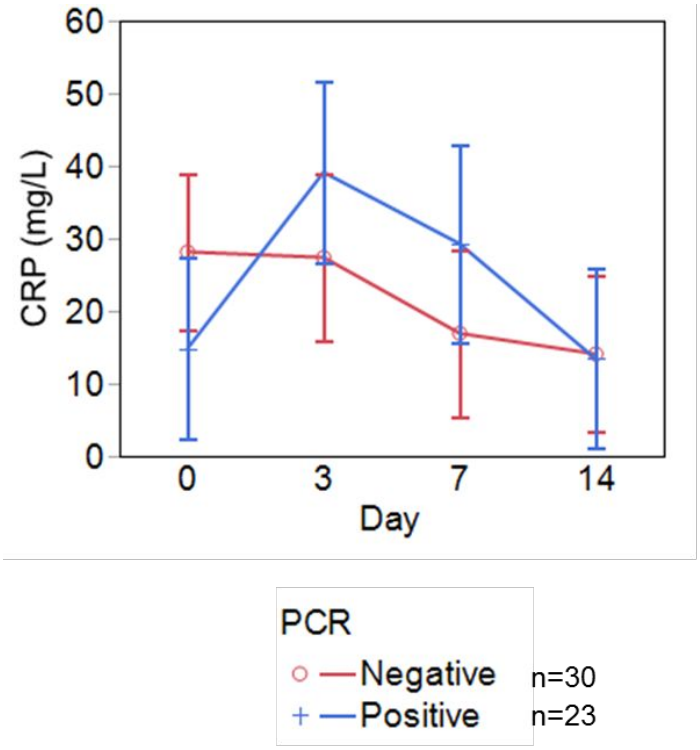

Figure S2: Serum Amyloid A reponse to PRRSV vaccine of pigs with negative and positive PCR results. Top panels, immunoenzymatic data; Other panel, mass spectrometry data from validated peptides. Error bars represent standard error of the mean.

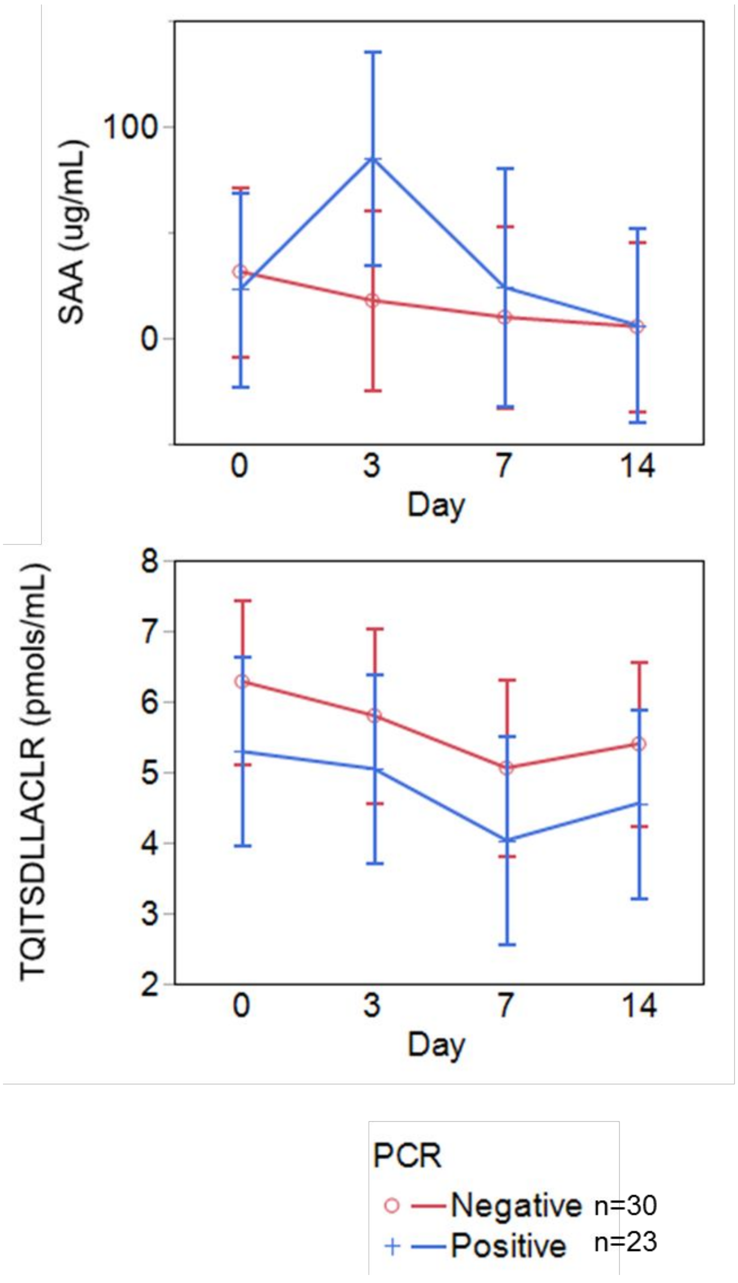

Figure S3: Apolipoprotein reponse to PRRSV vaccine of pigs with negative and positive PCR results. Mass spectrometry data from validated peptides. Error bars represent standard error of the mean.

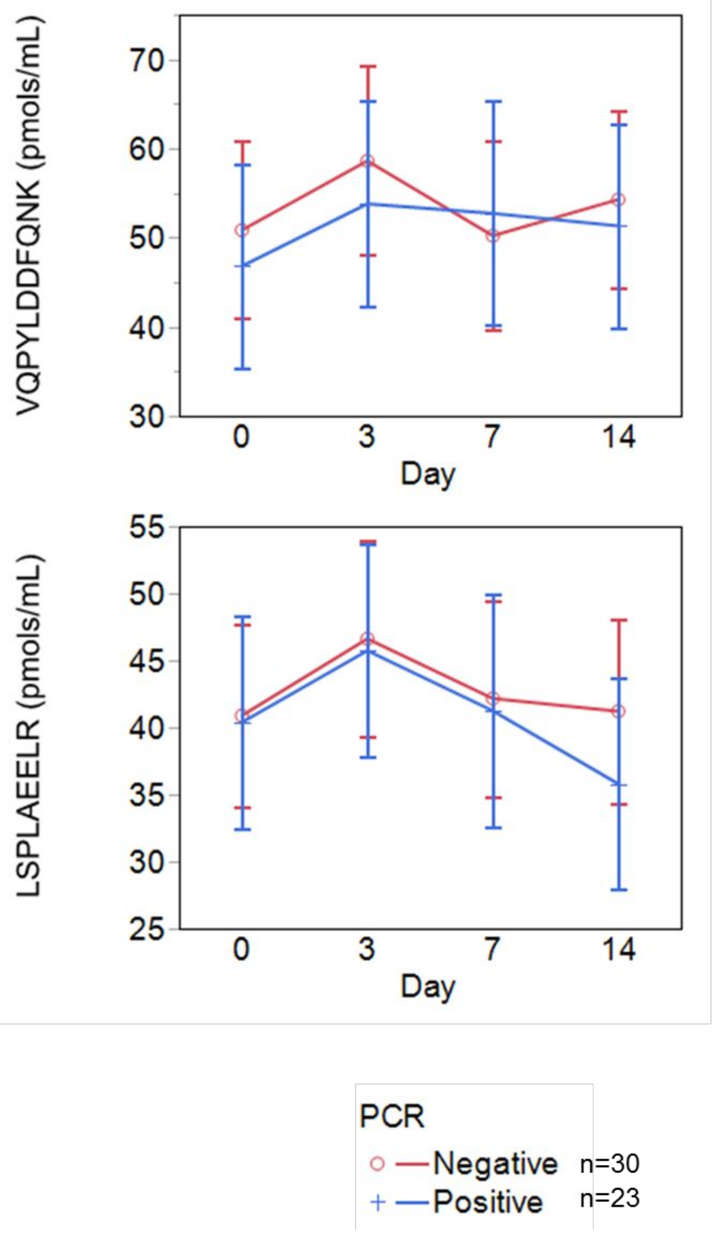

Figure S4: Distribution of the coefficients of variation of the 12 validated peptides. Three blood samples from the same pig have been used, obtained on 0, 3 and 14 of sampling days respectively. Each sample was analyzed in quintuplicate.

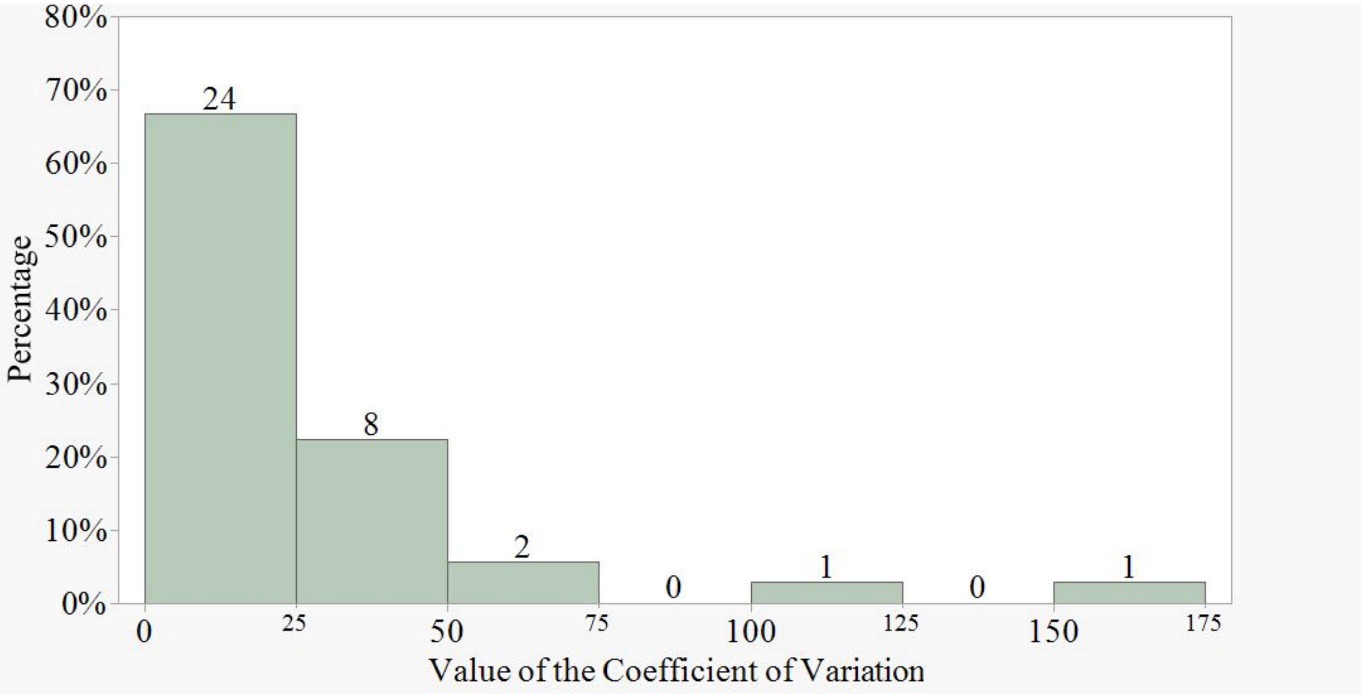

Supplement: Supplementary file 1 — pr4c00154_si_001.pdf [file pr4c00154_si_001.pdf]
